# Effects of Gas–Surface Interaction Conditions on the Performance of Knudsen Force-Based, Low-Pressure Micro Hydrogen Sensors

**DOI:** 10.3390/mi16050593

**Published:** 2025-05-19

**Authors:** Yanli Wang, Xiaowei Wang, Chunlin Du, Zhijun Zhang

**Affiliations:** 1 School of Mechanical Engineering, Ningxia Institute of Science and Technology, Shizuishan 753000, China; wangyl616@163.com; 2 School of Mechanical Engineering, Guangdong Ocean University, Zhanjiang 524088, China; 3 School of Mechanical Engineering and Automation, Northeastern University, Shenyang 110819, China; 4 Beijing Institute of Spacecraft Environment Engineering, Beijing 100094, China; to_cl2004@126.com

**Keywords:** thermally induced flow, Knudsen force, rarefied gas, DSMC, MEMS hydrogen sensors

## Abstract

Knudsen force phenomenon caused by non-uniform temperature fields in rarefied gas has been a topic of interest among researchers of gas sensing and structure actuating for micro-electromechanical systems (MEMS). The effects of gas–surface interaction conditions (accommodation coefficients, temperature differences, and carrier gases) on gas flows and hydrogen detection performance (Knudsen force) in MEMS gas sensors, consisting of a series of triangular cold beams and rectangular hot beams, are studied by using direct simulation Monte Carlo (DSMC) method combined with the Cercignani–Lampis–Lord (CLL) model in this work. The research results reveal that Knudsen force strongly depends on accommodation coefficients, temperature difference, and carrier gases. Specifically, the dependence of Knudsen force on accommodation coefficients is stronger at high pressure than at low pressure. In particular, Knudsen force increases slightly as accommodation coefficients are reduced from 1 to 0.1 but dramatically rises when accommodation coefficients verge on 0. In addition, Knudsen force is almost a linear function of temperature difference. The peak value of Knudsen force can be increased by roughly 28 times when the temperature difference rises from 10 K to 300 K. Last but not least, the linear correlation of hydrogen concentration in binary gas mixtures with Knudsen force is proposed for gas concentration detection in practice.

## 1. Introduction

Hydrogen sensors are widely applied in the fields of biomedical applications, clean energy, and nuclear industries for the detection of hydrogen concentration in devices or systems [1,2]. Certain gas sensors have problems of complicated structures, large volumes, and high manufacturing costs though various high-quality sensors have existed so far [2,3]. In order to break through the limitations, researchers have proposed a new method for gas detection based on Knudsen force effects [4,5,6,7,8,9]. Knudsen force effects are a peculiar physical phenomenon in rarefied gas environments (Knudsen number Kn ≳ 0.1). Specifically, directional gas flows are induced by directly or indirectly imposing thermal gradients or temperature differences on walls without initial pressure gradients in low-pressure environments or micro-electromechanical systems (MEMS) [10,11]. For example, for one of the most noted flows, thermal creep flows [11], over the walls with temperature gradients, when the fast hot molecules and slow cold molecules impinge on walls, net tangential momentum $F_{W}$ in the direction of temperature gradient is transferred to walls. The reaction force formed by $F_{W}$ drives the gas flows in the opposite direction of the temperature gradient, from which thermal creep flows are generated. On the other hand, there are significant differences in the momentum exchanged in the process of collisions of cold and hot molecules with walls, therefore exerting a net force on the solid structure, i.e., Knudsen force [12]. The value of Knudsen force is closely related to gas species and concentration, which can be applied to gas detection [4,5,6,7,8,9]. It is noted that compared with the conventional systems, MEMS has significantly enlarged surface area–volume ratios. Therefore, gas–surface interaction has significant effects on detection performance as well as on the characteristics of rarefied gas inside of MEMS hydrogen sensors based on Knudsen force.

A large number of studies have revealed that the interactions of gas–surface molecules are mainly determined by factors such as surface roughness, surface material, surface temperature, and gas species [13,14,15]. It is demonstrated that surface roughness has a significant effect on the characteristic behavior of friction factor, mass flow, heat transfer, flow patterns, flow velocity, and compression ratio by using experimental and numerical analysis methods [16,17,18,19,20,21]. In particular, surface roughness is usually modeled as a series of tiny obstacles uniformly distributed along the ideal smooth surface in numerical investigations. Moreover, the geometric shapes, structure parameters, and spacing of the obstacles can significantly impact the rarefied gas flow characteristics [19,20,21].

In physical reality, apart from surface roughness, wall conditions are closely related to surface material. Therefore, surface roughness and surface material are usually decoupled and separately studied for the effects on flow fields [15,22]. Investigations into the effects of surface material can be conducted by employing the common gas–surface interaction models (specular reflection, diffusive reflection, as well as the Maxwell [23] and Cercignani–Lampis–Lord (CLL) models [24,25]) and varying the accommodation coefficients of the model [22,23,24,25,26,27,28,29,30,31,32]. For example, Baier et al. [26,27] imposed specular reflection and diffuse reflection conditions on both sides of the plate, respectively, to achieve the applications of gas pumping and gas separation driven by temperature gradients. Based on the Maxwell model, by setting accommodation coefficients of the vertical and inclined walls of channels at different values, significant changes can be observed in the gas flow characteristics, including the increase in pumping speed and the reversal of pumping direction [33,34].

However, a large number of experimental and numerical results present that the CLL model, with two independent accommodation coefficients, provides many more correct physical descriptions of gas transport phenomena than the Maxwell model [35,36,37]. Therefore, the CLL model is currently considered to be the most reliable gas–surface interaction model. For the CLL model, the effects of normal energy accommodation coefficient (NEAC) and tangential momentum accommodation coefficient (TMAC) on the gas flow characteristics and force acting on solid structures were studied, respectively. It is found that TMAC has a more significant effect on the flow fields in micro-channels than NEAC [15]. For micro-beam systems (actuators) with different temperatures, when only the accommodation coefficients of the cold beam are changed, Knudsen force on the cold beam increases approximately linearly with the decrease in NEAC, but decreases nonlinearly as TMAC is reduced [22]. On the contrary, Knudsen force on the cold beams decreases nonlinearly as NEAC decreases, but it increases nonlinearly, with TMAC reducing, when the accommodation coefficients of both cold and hot beams are simultaneously changed [30]. It is noted that the research on each cold beam surface separately configured with different accommodation coefficients has not been reported yet. For a sphere with uniform temperature, the force acting on the sphere always increases with the increase in NEAC, but the qualitative effect of TMAC on the force depends on rarefaction parameters [31]. In addition, the value of the accommodation coefficient is also related to the gas species. For example, for a variety of smooth metal (aluminum, platinum, and stainless steel) surfaces, the NEAC for helium is between 0 and 0.1, but ranges from 0.5 to 0.95 for argon; the TMACs for helium and argon are both between 0.5 and 1 [38,39].

On the other hand, the velocity of gas molecules reflected from a solid surface depends on the gas species and the surface temperature. More specifically, the molecular velocity is inversely proportional to the square root of the molecule mass and directly proportional to the square root of the surface temperature. Therefore, the gas species and the surface temperature have a significant influence on the gas flows (thermal creep flow, thermal edge flow, radiometric flow, etc.) induced by the peculiar thermal effects near the boundary in the rarefied gas, as well as the force exerting on the solid structure. For example, as the temperature difference between adjacent surfaces of the ratchets increases, the degree of gas separation increases nonlinearly [40]. Within a small temperature difference range, the mass flow rate increases approximately linearly as the temperature difference between the cold and hot walls rises [41]. However, over large temperature difference ranges, the dependence of mass flow rate on temperature difference is nonlinear [34,41,42]. For Knudsen force, as temperature of the immersed structure increases, the maximal value of the force is obtained under higher ambient pressure [9]. Under the condition that the temperature difference between the cold and hot beams increases from 10 K to 100 K, the maximal value of Knudsen force can be increased by more than 7 times [43]. In addition, through investigating Knudsen force exerted on solid structures in different carrier gas atmospheres, it can be observed that the values of Knudsen force also depend on the diameter of gas molecules [44,45,46,47]. That is, Knudsen force increases as the diameter of the carrier gas molecule decreases. For binary gas mixtures, the Knudsen force is a linear function of any species concentration, and the line slope is related to the molecular diameter and ambient pressure [46,47].

Considering the background mentioned above, the performance of hydrogen detection of MEMS sensors composed of rectangle hot beams and triangle cold beams is assessed in the present work. Compared to the rectangular hot–cold beam configuration in previous studies, beams or blades with sharp edges can enhance the sensor operation performance [48,49]. At the same time, Knudsen force characteristics can be further enhanced when different surface accommodation coefficients are imposed on the inclined top/bottom surfaces and vertical right surface of the cold beam. Currently, the main objective of this study is to numerically investigate the effects of some key parameters of gas–surface interaction, such as accommodation coefficients of beam surfaces, temperature difference of the cold and hot beams, carrier gas, and Knudsen numbers that determine the gas flow scale in sensors. The remaining part of this paper is structured as follows: Section 2 presents the configuration of MEMS hydrogen sensors composed of triangular cold beams and rectangular hot beams. Section 3 describes the direct simulation Monte Carlo (DSMC) method applied to the research, relevant modeling assumptions, and calculation parameters. In Section 4, DSMC simulation results for different factors are given and analyzed. Lastly, conclusions of the work are presented in Section 5.

## 2. Problem Statement

**Figure 1:**
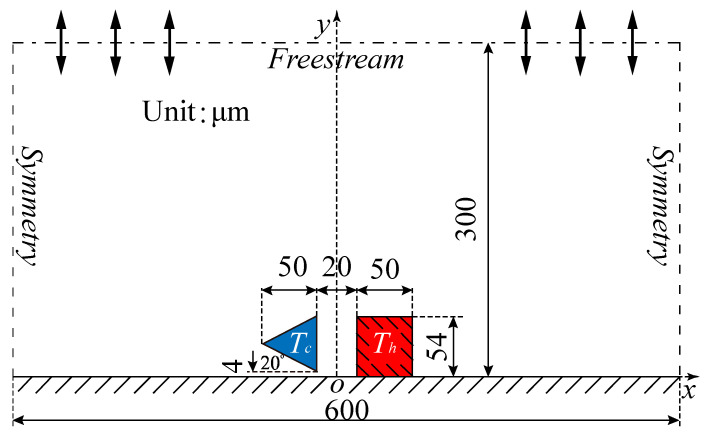
Computational domain and boundary conditions used for DSMC simulation setup.

Figure 1 schematically illustrates the MEMS hydrogen sensor configuration consisting of a series of rectangular hot beams and triangular cold beams. The cross-section dimension of the triangular cold beam at $T_{c}$ is $(w\;\times \;h)/2\;\mu m^{2}$, and the angle between the bottom surface and −x direction is β=20°. w and h are the width and height of the cold beam, respectively. The rectangular hot beam, at $T_{h}$, having a G=20 μm distance from the cold beam, has a cross-section dimension of $w\;\times \;(h+4)\;\mu m^{2}$. In the research, only the force acting on the cold beam is considered. Thus, the hot beam is fixed to the substrate, while the cold beam is suspended 4 μm above the substrate. Wall boundary condition is applied to the substrate, with wall temperature $T_{s}=298\;K$. In order to improve the simulation efficiency, only one cold–hot beam pair is investigated within the domain of $W\times H=600\times 300\;{\mu m}^{2}$ for the gas flows and force characteristics. Moreover, the beam length in z direction is considered to be much larger than the cross-section dimension. Therefore, two-dimensional DSMC simulations are carried out.

In the DSMC simulations, symmetric boundary conditions were imposed on the left and right sides of the computational domain, as illustrated in Figure 1. When simulation particles hit the symmetric boundaries, specular reflection occurs and the particles come back to the domain. Freestream boundary conditions ($T_{0}=296\;K$) with parameters of the initial gas mixtures are imposed on the upper side of the domain. The simulated particles can flow in or out the domain through freestream boundary.

In this work, the reference configuration parameters are used when no specific descriptions of the factors are given. That is, (i) accommodation coefficients TMAC ($\sigma_{t}$) and NEAC ($\alpha_{n}$) of all the walls are set as 1; (ii) carrier gas is the binary gas mixtures consisting of 50% H_2_ and 50% N_2_. Moreover, in order to be physically practical (to increase Knudsen force), only the accommodation coefficients of the cold beam-inclined walls (the top and bottom surfaces) ($\sigma_{t}\;=\;\alpha_{n}\;=\;\alpha$) vary in the range of 0 ≤ α ≤ 1, while the accommodation coefficients of the vertical right surface of the cold beam and all the surfaces of the hot beam remain unchanged (α = 1).

## 3. Numerical Method and Computation Parameters

In this part, the DSMC method and the corresponding open-source solver used in the research are first introduced. Then, schemes of the relevant modeling assumptions for the DSMC simulations are presented. Finally, information on varieties of parameters for the DSMC simulations is described in detail.

### 3.1. DSMC Method and Solver

The powerful DSMC method can numerically solve the problems of rarefied gas flows [50]. In order to simulate gas flows, a large number of simulated particles are used to represent real gas molecules. In DSMC simulations, the free movement of particles and the collisions between particles are broken down into two separate parts within a time step (Δt) much smaller than the mean collision time. In the process of free motion of particles, each particle moves at the molecule velocity in space. The particles can stay inside the same cell, move to the adjacent cells, be reflected from walls or symmetric boundaries, or leave the computation domain through outflow boundary. Indexing is then conducted according to the new position of each particle and the new cell in which it is located. Thus, the changes in position, velocity, and energy caused by inter-particle collisions and collisions of particles with boundary surfaces are calculated. Finally, the macroscopic flow characteristics can be obtained from the simple weighting averages of microscopic characteristics. Note that enough time steps are iterated to reduce the statistical error, so as to obtain the real gas flow characteristics.

An open-source DSMC solver, dsmcFoamPlus, is adopted [51]. The reliability of the solver has been widely verified in various studies of rarefied gas flows, with thermally driven flows included [40,41,42,43,44,45]. Given that the working fluid comprises binary gas mixtures of H_2_-N_2_, the Larsen–Borgnakke variable soft sphere collision model is applied for the inter-particle collisions in DSMC simulations. In addition, the standard no-time-counter collision scheme is employed for the selection scheme of collision pairs. As mentioned above, in order to study the influence of accommodation coefficients, all wall surfaces are set as a CLL model, which is used to describe the interaction of gas molecules with solid surfaces.

### 3.2. Computation Parameters

Table 1 displays temperatures of hot and cold beams at different pressures. It is noted that the values of temperature are obtained from averaging the experimental values calculated in the pure N_2_ atmosphere [4,5] at the corresponding pressures. Moreover, with G as the characteristic length of the beam system, the obtained Kn of each gas species and gas mixtures are also presented in Table 1. The calculation of Kn is shown as follows [50]:

(1) $$Kn\;=\;\frac{\lambda }{G}\;=\;\frac{\sum_{i=1}^{2}C_{0i}\lambda_{i}}{G},$$

(2) $$\lambda_{i}\;=\;{\left(\frac{\pi P_{0}}{4k_{B}T_{0}}\sum_{j=1}^{2}C_{0j}{\left(d_{i}\;+\;d_{j}\right)}^{2}\sqrt{1+m_{i}/m_{j}}\right)}^{-1},$$

where $C_{0i}$ and $C_{0j}$ denote the initial mole fractions of gas species i and j, respectively. $P_{0}$ represents the initial pressure, $k_{B}$ is the Boltzmann constant, $T_{0}$ is the initial temperature, and d and m denote diameter and mass of the molecule, respectively.

In order to analyze the effects of surface material, different values of $\sigma_{t}$ and $\alpha_{n}$ are considered. With $0\;\leq \;\alpha =\sigma_{t}\;=\;\alpha_{n}\;\leq \;1$, $\alpha =\sigma_{t}\;=\;\alpha_{n}\;=\;0$ corresponds to the specular reflection at the solid surface, and $\alpha =\sigma_{t}\;=\;\alpha_{n}\;=\;1$ corresponds to the diffuse reflection at the solid surface. The effects of temperature difference ΔT between the cold and hot beams are investigated using nine cases with $10\;K\;\leq \;\Delta T\;=T_{h}\;-\;T_{c}\;\leq \;300\;K$. Finally, the influence of carrier gases is analyzed by changing the equilibrium mole concentration $C_{0}$ of H_2_ in the gas mixtures. With $C_{0}\;=\;n_{0}^{H_{2}}/(n_{0}^{H_{2}}+n_{0}^{N_{2}})$, $n_{0}^{H_{2}}$ and $n_{0}^{N_{2}}$ denote the equilibrium number densities of H_2_ (the lighter gas) and N_2_ (the heavier one), respectively. Two limits, $C_{0}\;\to \;0$ and $C_{0}\;\to \;1$, correspond to pure N_2_ and H_2_ atmospheres, respectively.

For numerical parameters of DSMC, in all simulations, the computational domain is discretized according to the maximal cell size of Δx = Δy = 2 μm. More specifically, each cell includes roughly 20 particles on average. Δt is considerably shorter than the mean collision time, with a constant value of Δt = 1 ns. Each DSMC simulation runs for at least 7 ms so as to decrease the statistic error. In addition, the independence tests for cell size, particles per cell (PPC), Δt, and total simulation time (t) are exhibited in detail in Section 4.1.

## 4. Results and Discussion

### 4.1. Independence Tests for Solution Parameters

It is well known that cell size, PPC, Δt, and t can influence the accuracy of DSMC results. Therefore, the validation investigations into the four numerical parameters with the reference configuration at the largest pressure and temperature, i.e., $P_{0}\;=\;966\;\mathrm{Pa}\;(Kn\;=\;0.371)$ and ΔT = 300 K, are carried out.

**Figure 2:**
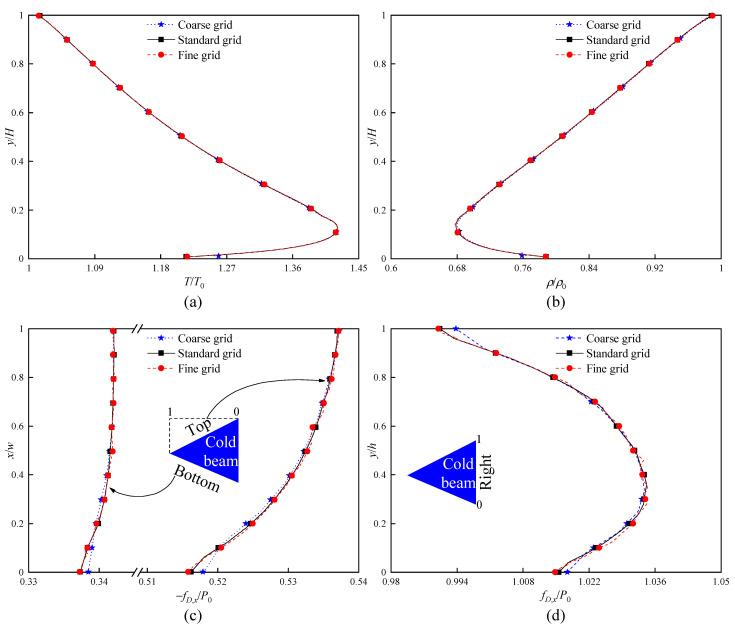
Grid dependence checks (PPC = 20, Δt = 1 ns), with coarse, standard, and fine grids corresponding to 10,942, 44,470, and 177,880 cells, respectively, at Kn = 0.371. (**a**) Temperature distributions over a vertical line at x = 0. (**b**) Density distributions over a vertical line at x = 0. (**c**) Force distributions along the top and bottom surfaces of the cold beam. (**d**) Force distributions along the right surface of the cold beam.

Firstly, the dependence checks for three cell sizes are conducted, that is, 10,942 cells (coarse grids), 44,470 cells (standard grids), and 177,880 cells (fine grids). Figure 2 illustrates the flow field characteristics (temperatures and density) over the vertical line at x = 0 and the force distributions along the cold beam surfaces obtained for three different grids. It is found that the results of the standard and fine grids are slightly different, while the difference between these two grids and the coarse grid results is significantly different. Therefore, it is indicated that the application of standard grids is sufficient to provide solutions independent of the grids.

**Figure 3:**
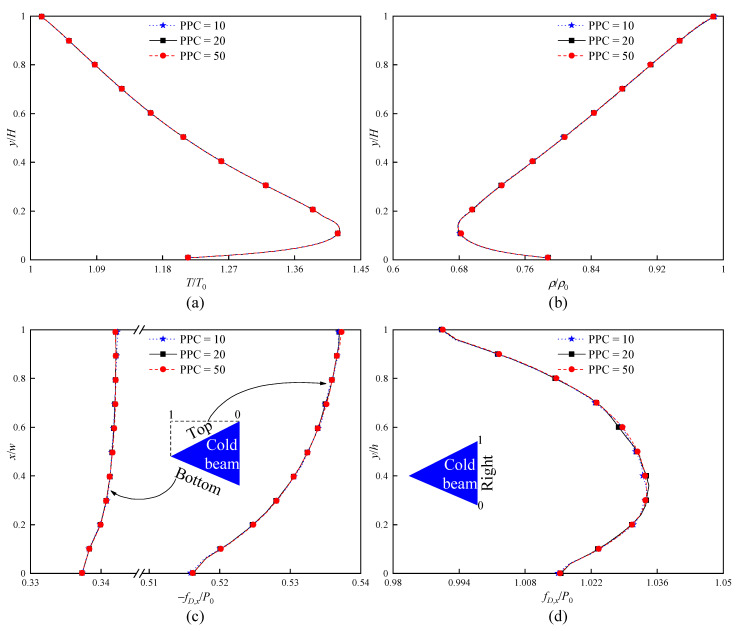
PPC dependence checks (standard grid, Δt = 1 ns) at Kn = 0.371. (**a**) Temperature distributions over a vertical line at x = 0. (**b**) Density distributions over a vertical line at x = 0. (**c**) Force distributions along the top and bottom surfaces of the cold beam. (**d**) Force distributions along the right surface of the cold beam.

Secondly, the dependence tests for PPC are performed with the standard grids, that is, PPC = 10, 20, and 50. The obtained temperature, density, and force distributions based on the three PPCs are illustrated in Figure 3. It can be found that the difference in the results for three PPCs is almost negligible. Therefore, it is assumed that the simulation results independent of PPC can be sufficiently provided at PPC = 20.

**Figure 4:**
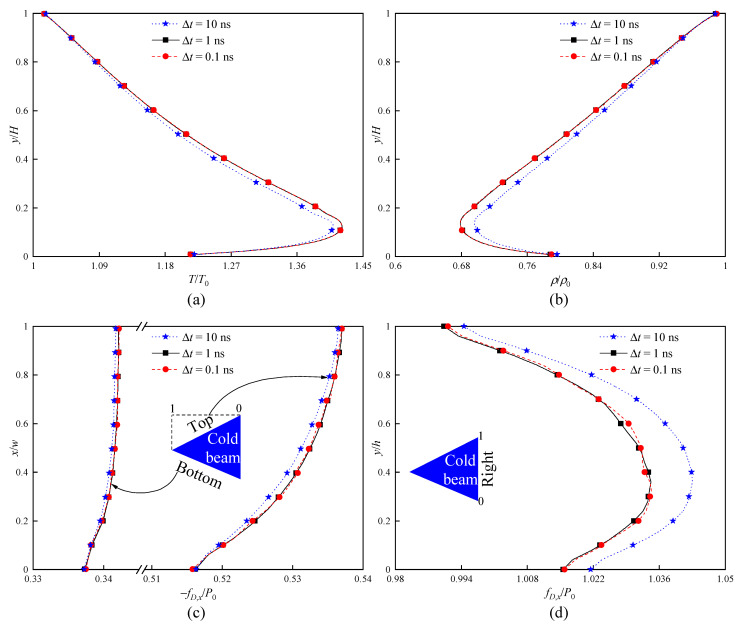
Time step dependence checks (standard grid, PPC = 20) at Kn = 0.371. (**a**) Temperature distributions over a vertical line at x = 0. (**b**) Density distributions over a vertical line at x = 0. (**c**) Force distributions along the top and bottom surfaces of the cold beam. (**d**) Force distributions along the right surface of the cold beam.

In addition, the influence of time step Δt is tested based on the numerical parameters of standard grids and PPC = 20. Compared with the basic case of time step (Δt = 1 ns), two news cases of Δt are separately reduced and increased by 10%, i.e., Δt = 10 ns and Δt = 0.1 ns. Figure 4 illustrates the temperature, density, and force distributions for three different Δt. It can be found that the difference between the results of the cases of Δt = 0.1 ns and Δt = 1 ns is almost negligible, while the results of the case of Δt = 10 ns significantly differ from data of the cases of Δt = 0.1 ns and Δt = 1 ns. This reveals the strong dependence of flow fields and force characteristics on time step Δt. For the current issue, the application of Δt = 1 ns to the calculation of field characteristics is sufficient.

**Figure 5:**
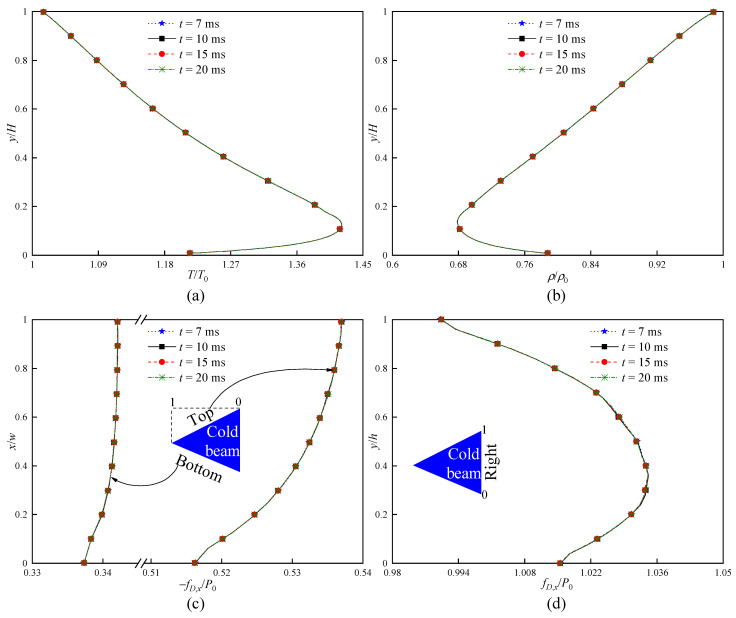
Total simulation time dependence checks (standard grid, PPC = 20, Δt = 1 ns) at Kn = 0.371. (**a**) Temperature distributions over a vertical line at x = 0. (**b**) Density distributions over a vertical line at x = 0. (**c**) Force distributions along the top and bottom surfaces of the cold beam. (**d**) Force distributions along the right surface of the cold beam.

Lastly, the influence of the total simulation time t on the result stability is checked by applying the numerical parameters of standard grids, PPC = 20, and Δt = 1 ns. Four t are considered, i.e., 7 ms, 10 ms, 15 ms, and 20 ms. Figure 5 presents the temperature, density, and force distributions for the four different t. It is revealed that the difference in the results for the four t is almost negligible. This means that, after the cases run for t ≥ 7 ms, the results can basically reach a stable condition. Moreover, the errors of the predicted flow characteristics are reduced to the acceptable level.

In addition, the results of the average flow field characteristics over a vertical line at x = 0 and Knudsen force on the cold beam for different cell sizes, PPCs, Δt, and t are reported in Table 2 and Table 3. All these figures and tables clearly exhibit that the dependence of flow fields and force characteristics on the numerical parameters are almost negligible within the considered ranges of cell sizes (standard and fine grids), PPC ≥ 10, Δt ≤ 1 ns, and t ≥ 7 ms. The computations of DSMC solution schemes become more costly for small cell size and Δt, as well as large PPCs. Therefore, the numerical parameters of standard grid (44,470 cells), PPC = 20, Δt = 1 ns, and t = 10 ms are applied to all DSMC calculations in the current research.

### 4.2. Effects of Accommodation Coefficients

In this part, the influence of the surface material is investigated by varying the accommodation coefficients of cold beam-inclined top and bottom surfaces, with the geometry and position of the beams remaining unchanged.

**Figure 6:**
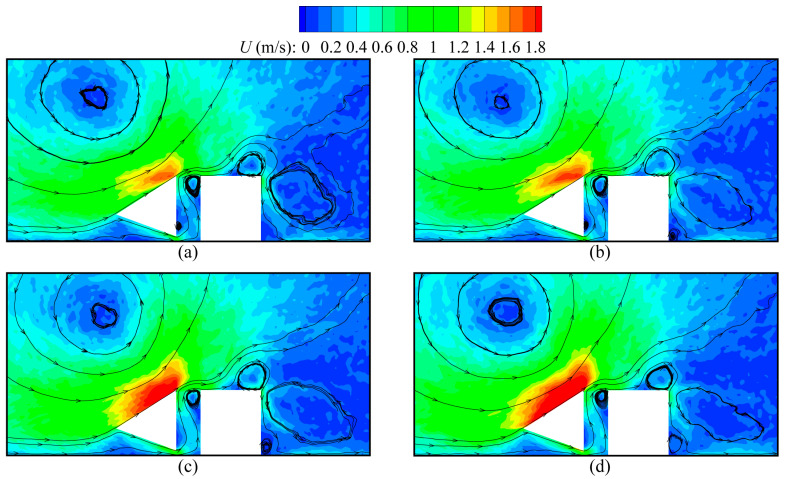
Speed contours and velocity streamlines around the cold–hot beam pair for different values of accommodation coefficient α, at Kn = 0.926. (**a**) α = 0.8, (**b**) α = 0.6, (**c**) α = 0.2, (**d**) α = 0.

The speed contours and velocity streamlines around beams for various values of the accommodation coefficient α are illustrated in Figure 6. As expected, the gas flow characteristics strongly depend on α. The temperature gradients near the inclined walls increase owing to the decrease in α, which is more effective to induce gas flows. Thus, the overall speed of gas flows significantly increases as α reduces. This qualitative behavior is similar to the results reported in Ref. [29]. In addition, it is found that the difference in flow patterns due to the change in α only appears near the cold beam lower tip. That is, with α reducing, the size of the vortex near the cold beam lower tip decreases and even disappears, because the stronger rightward flows occur for a small α. However, different from the current results, the previous research reported that α causes a big difference in the flow patterns [30]. In fact, in Ref. [30], the accommodation coefficients of all surfaces vary, whereas only the accommodation coefficients of the cold beam-inclined walls are changed in the current work.

Knudsen force, an important parameter for the current cold–hot micro-beam hydrogen sensors, can be obtained from the simulation. In DSMC, the force density $f_{D}$ acting on the solid walls can be obtained from the incident and reflected the momentum of molecules colliding with surfaces per unit area, is expressed as follows:

(3) $$f_{D}=\frac{F_{N}}{dA\Delta t}\sum_{j=1}^{N}m_{j}(c_{j}^{r}-c_{j}^{i}),$$

where $F_{N}$ denotes the real number of gas molecules denoted by one simulation particle, dA represents the unit area of particles impinging on walls, N indicates the number of simulation particles colliding with walls per unit area dA within time step Δt, m is the mass of simulation particles, c is the velocity of simulation particles, the superscripts i and r represent incident and reflected particles, respectively.

The cold beam is assumed to move only in x direction; thus, only the force density of x component $f_{D,x}$ on the cold beam is considered. It is noted that force density $f_{D,x}$ in x direction leads to the attraction between cold and hot beams, while $f_{D,x}$ in −x direction causes the repulsion between cold and hot beams. Therefore, $f_{D,x}$ in x and −x directions are considered to be negative and positive, respectively. Lastly, the net Knudsen force exerting on a cold beam can be obtained from the integration of the force density on all surfaces of the cold beam.

**Figure 7:**
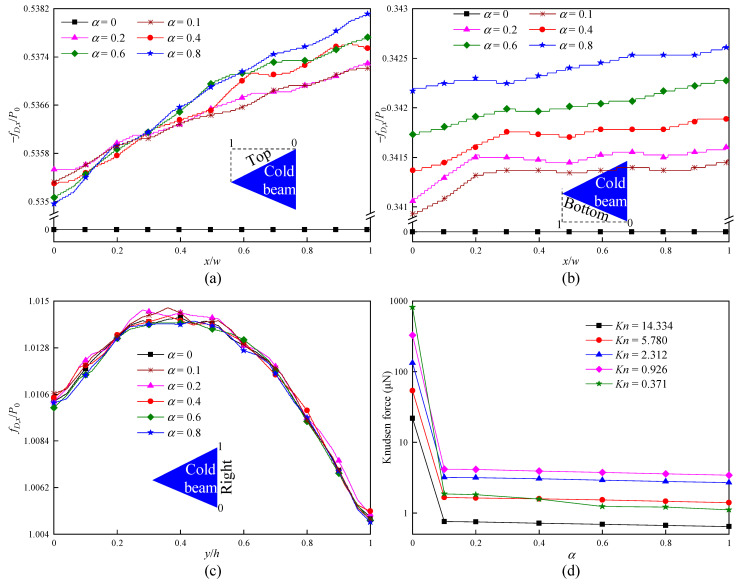
Force on the cold beam for different accommodation coefficients α. (**a**) Force distributions on the top surface at Kn = 0.926. (**b**) Force distributions on the bottom surface at Kn = 0.926. (**c**) Force distributions on the right surface at Kn = 0.926. (**d**) Knudsen force.

In order to quantitatively analyze the influence of α on the force exerting on a cold beam, Figure 7 illustrates the dimensionless force density $f_{D,x}/P_{0}$ distributions over all cold beam surfaces and the net Knudsen force. Similar to the results reported in Refs. [29,30,31,32], Figure 7 exhibits a strong dependence of force on α. For the top surface, when α reduces, the overall variation tendency of $f_{D,x}/P_{0}$ decreases. This is completely correct, because a small α means less molecules exchanging momentum with the wall. However, it is found that, in the region close to the cold-beam upper tip (x/w ∼ 0), $f_{D,x}/P_{0}$ slightly increases when α reduces from 0.8 to 0.2, as illustrated in Figure 7a. The reason appears to be that, for a small α, the transferred momentum of the fast molecules with higher temperature impinging on the wall can offset the momentum of less molecules exchanging with the wall. Compared with the situations on the top surface, the phenomenon of less molecules exchanging momentum with the wall dominates the bottom surface. This is also the reason for the fact that $f_{D,x}/P_{0}$ significantly decreases as α reduces, as presented in Figure 7b. Note that, at α = 0, $f_{D,x}/P_{0}\;∼\;0$ for both the top and bottom surfaces occurs. This can be expected, because all molecules impinging on the surfaces perform specular reflection, i.e., no momentum or energy exchanged between the gas molecules and the walls. On the other hand, from Figure 7c, it is observed that the difference in $f_{D,x}/P_{0}$ distributions on the right surface for different α of the inclined walls is almost negligible. This means that the force exerting on the wall heavily depends on α of the wall itself, while the influence of α on the other walls is negligible.

As expected, the Knudsen force depends nonlinearly on α, and it increases as α reduces, as illustrated in Figure 7d. In particular, when α decreases from 1 to 0.1, the magnitude of the increase in Knudsen force is small, while Knudsen force rapidly increases when α verges on zero. This means that using special material or treating the surfaces differently to make α verge on zero is extremely important to maximize Knudsen force. The results also demonstrate that, in a high-pressure environment, the dependence of Knudsen force on α is stronger than that in a low-pressure environment. Quantitatively, when α varies from 1 to 0, Knudsen force increases by approximately 34 times at Kn = 14.334, but rises by approximately 736 times at Kn = 0.371. The similar qualitative behavior is reported in Refs. [29,30,31,32]. In fact, within the Kn range for the generation of Knudsen force, the behavior is triggered by the fact that a higher pressure corresponds to a higher gas density; thus, more molecules interact with the walls.

Therefore, changing surface accommodation coefficients through the application of different material or different treatments on surfaces can be an effective way to adjust the value of Knudsen force. Table 4 presents the effects of surface accommodation coefficients of each beam on Knudsen force from the current research and the previous literature.

### 4.3. Effects of Temperature Difference

Here, the effects of work conditions on the gas flows and Knudsen force are analyzed. The temperature difference ΔT between the cold and hot beams is an important factor, because temperature is the energy source to induce the rarefied gas flows in the current issue. The influence of ΔT is investigated at varying the hot beam temperature $T_{h}$, with all other parameters remaining at the reference conditions. In practice, the variation in temperature can be achieved by simultaneously increasing the power of the hot beam heating elements and enhancing the cooling efficiency of the cold beam.

**Figure 8:**
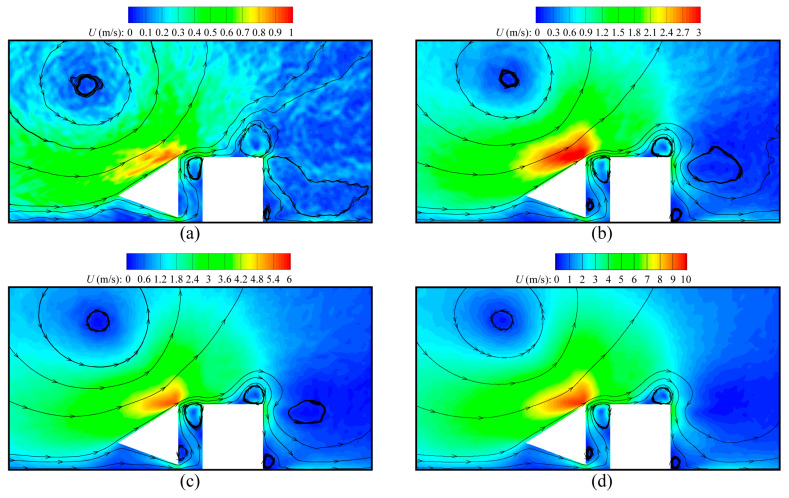
Speed contours and velocity streamlines around the cold–hot beam pair for different values of temperature difference ΔT at Kn = 0.926. (**a**) ΔT = 20 K, (**b**) ΔT = 80 K, (**c**) ΔT = 150 K, (**d**) ΔT = 300 K.

Figure 8 illustrates the speed contours and velocity streamlines around beams for ΔT = 20 K, 80 K, 150 K, and 300 K at Kn = 0.926 and $T_{c}\;=\;304\;K$. As expected, ΔT has a strong effect on the gas flows inside the device. The overall speed of the gas flows significantly increases as ΔT increases, which can be expected as a large ΔT corresponds to stronger temperature gradients, i.e., a larger inducing velocity. Likewise, compared with the situations for a low temperature gradient (ΔT = 20 K), the streamline patterns are clearer in the case of a high temperature gradient (ΔT = 300 K), as illustrated in Figure 8d. This similar qualitative behavior can be found in the previous literature [43]. On the other hand, as ΔT rises, the sizes of the vortices near the hot beam upper-right tip decrease, and one of the vortices even disappears owing to the competitive effect between the thermal edge flows and the rightward flows. Although the flows are enhanced by the increase in ΔT, the rightward flows depend more on the temperature gradients than the thermal edge flows. That is, with ΔT increasing, the contribution of the rightward flows to the overall gas flows within the device becomes more dominant.

**Figure 9:**
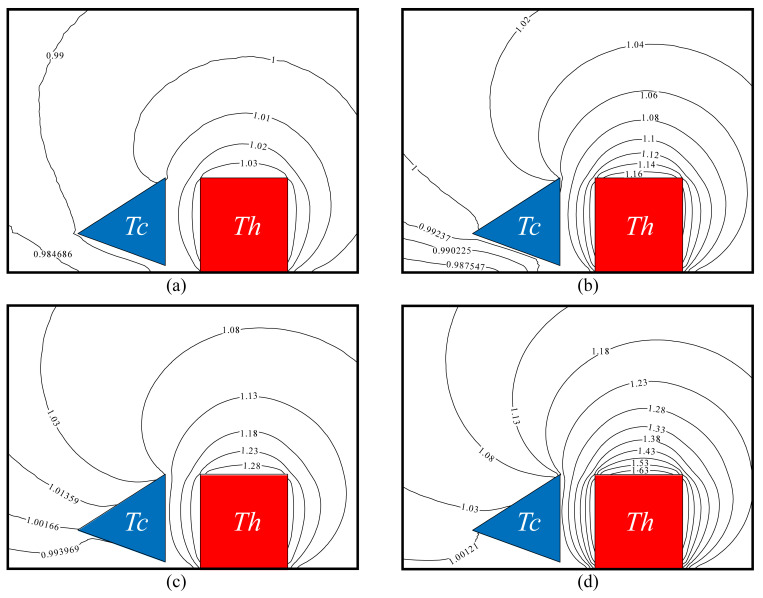
Isolines of normalized temperature around the cold–hot beam pair for different values of temperature difference ΔT at Kn = 0.926. (**a**) ΔT = 20 K, (**b**) ΔT = 80 K, (**c**) ΔT = 150 K, (**d**) ΔT = 300 K.

Figure 9 illustrates the close-up views of the normalized temperature $T/T_{c}$ contours around the beams for several representative cases corresponding to ΔT = 20 K, 80 K, 150 K, and 300 K at Kn = 0.926 and $T_{c}\;=\;304\;K$. It can be observed that temperature contours change dramatically for different ΔT. As expected, the values of isolines increase as ΔT increases. For all cases, the isolines far from the tip regions of beams are generally uniform, while the isolines near the beam tips change sharply. In addition, the isolines near the beam tips vary more dramatically as ΔT increases. It is demonstrated that the increase in ΔT can trigger stronger thermal edge flows.

**Figure 10:**
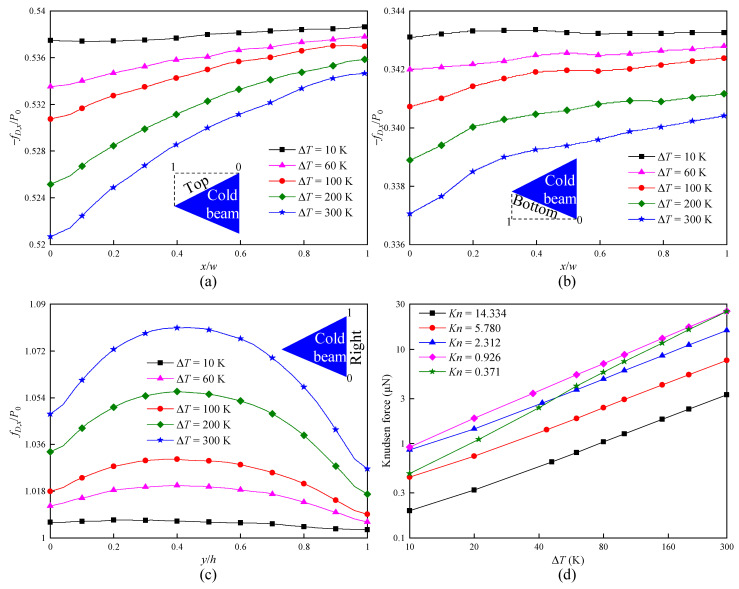
Force on the cold beam for different temperature differences ΔT. (**a**) Force distributions on the top surface at Kn = 0.926. (**b**) Force distributions on the bottom surface at Kn = 0.926. (**c**) Force distributions on the right surface at Kn = 0.926. (**d**) Knudsen force.

In order to quantitatively analyze the influence of ΔT on the force exerting on the cold beam, Figure 10 presents the dimensionless force density $f_{D,x}/P_{0}$ distributions over all cold beam surfaces and the net Knudsen force. It can be observed that, when ΔT rises from 10 K to 300 K, the variations in $f_{D,x}/P_{0}$ on the inclined surfaces and the vertical surface behave totally differently. For example, for the inclined top and bottom surfaces, both $f_{D,x}/P_{0}$ significantly decrease as ΔT rises, as illustrated in Figure 10a,b. In contrast, for the vertical right surface, $f_{D,x}/P_{0}$ significantly increases with the increase in ΔT, as illustrated in Figure 10c. The difference lies in the following two facts: (i) Under the same pressure conditions, the gas density is smaller in gases with a high temperature. Moreover, the low-velocity molecules reflected from the cold beam hinder the collisions of new molecules around the cold beam. This means less molecules impinge on the surfaces and transfer momentum. (ii) The molecular velocity is directly proportional to square root of the gas temperature. The high-velocity molecules at high temperature collide with the low-velocity molecules reflected from the cold beam, and momentum is exchanged. In this way, the low-velocity molecules can impinge on the cold beam again at a larger velocity. The former (the first fact) dominates the generation of the force on the top and bottom surfaces, while the latter (the second fact) plays a dominant role in the force produced on the right surface. At the same time, the highest gas temperature along the surfaces corresponds to x/w = 0 (the bottom and top surfaces) and y/h ∼ 0.5 (the right surface); thus, the effects of ΔT on $f_{D,x}/P_{0}$ near the regions are the strongest.

As expected, Knudsen force increases with an increase in ΔT, as illustrated in Figure 10d. In fact, this qualitative behavior can be found in Ref. [43]. However, from Figure 10d, it is observed that Knudsen force is almost a linear function of ΔT. In particular, with Kn decreasing, the slopes of the Knudsen force curves obviously rise. It can be concluded that Knudsen force depends more on ΔT at high pressure than at low pressure. For example, when ΔT rises from 10 K to 300 K at Kn = 14.334, Knudsen force increases by approximately 17 times, while rising by roughly 53 times at Kn = 0.371. This is because more gas molecules impinge on the walls and transfer energy in a high-pressure environment. Therefore, in actual applications, a simple increase in heating input power of a hot beam can enhance Knudsen force, which leads to an improvement in the performance of sensors.

### 4.4. Effects of Species Concentration

**Figure 11:**
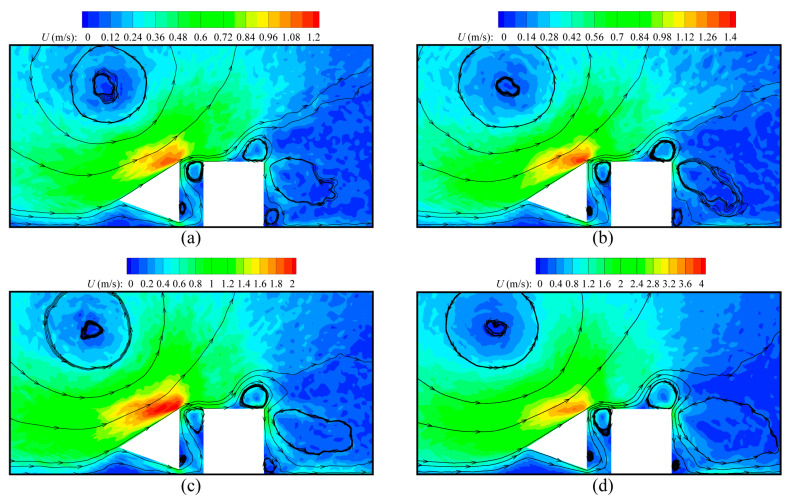
Speed contours and velocity streamlines around the cold–hot beam pair for different values of species concentration $C_{0}\;$at Kn = 0.926. (**a**) $C_{0}\;=\;0$, (**b**) $C_{0}\;=\;0.25$, (**c**) $C_{0}\;=\;0.75$, (**d**) $C_{0}\;=\;1$.

This type of device is designed for gas detection in practice; thus, the influence of gas compositions is investigated by varying the equilibrium mole concentration $C_{0}$ of H_2_ in the gas mixtures. Figure 11 illustrates the speed contours and velocity streamlines around the beams obtained by the DSMC method for different $C_{0}$. It is observed that the overall speed of gas flows has a significant dependence on $C_{0}$, and significantly increases as $C_{0}$ rises. The reason for this is that the molecular velocity is inversely proportional to the square root of the molecular mass; thus, the velocity of the lighter H_2_ is larger than that of the heavier N_2_. As a result, the overall speed of the H_2_-dominating mixtures is considerably higher than that of the N_2_-dominating mixtures. On the other hand, for any case of $C_{0}$, the flow patterns are the same. This means that the influence of $C_{0}$ on the gas flow patterns is negligible. The qualitative behavior was also reported in Refs. [46,47].

**Figure 12:**
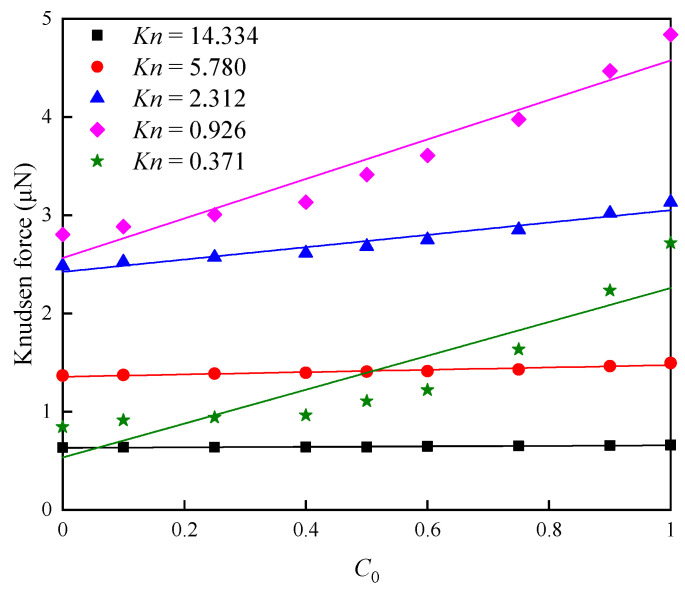
Knudsen force on the cold beam for different species concentrations $C_{0}$.

To quantitatively evaluate the effects of $C_{0}$ on Knudsen force performance, Figure 12 illustrates the function relation of $C_{0}$ to Knudsen force. It can be observed that, for all cases of Kn, the obtained Knudsen force strongly depends on $C_{0}$, increasing as $C_{0}$ rises. This result is similar to data reported in Refs. [46,47]. In fact, the molecular mass and the molecular diameter (or collision frequency) are the two main factors. As mentioned above, the first factor can change the molecular velocity; thus, the increase in $C_{0}$ increases the velocity of molecules impinging on the walls. The second factor can vary the number of molecules colliding with the walls, leading to the variations in Knudsen force. More specifically, the molecular diameter of H_2_ is substantially smaller than that of N_2_. Thus, the mean free path of H_2_ is larger. This means that, in the H_2_-dominating (a larger $C_{0}$) mixtures, more molecules impinge on the surfaces per unit time, and a larger Knudsen force is obtained.

In addition, from Figure 12, it can be found that the dependence of Knudsen force on $C_{0}$ is almost linear. Thus, it can be predicted that the linear correlation of Knudsen force with $C_{0}$ in N_2_-H_2_ mixtures can be applicable to the assessment of hydrogen concentration in practice. The expression is as follows:

(4) $$KF\;=\;F_{N_{2}}\;+\;SC_{0},$$

where $F_{N_{2}}$ represents the force generated in the pure nitrogen environment, $C_{0}$ denotes the concentration of hydrogen in the gas mixtures, and S is the slope for each line in Figure 12. Note that, when the diameter of the gas molecule is larger than that of nitrogen, the value of slope S is negative; otherwise, the value of S is positive [46,47]. Compared with the absolute values of S in Refs. [46,47], the value of S in this research is substantially larger. The reason appears to be the sharper edge of the current cold beam, resulting in stronger Knudsen force.

On the other hand, with $P_{0}$ increasing, the slopes of Knudsen force curves significantly increase. For example, when $C_{0}$ rises from 0 to 1, at Kn = 14.334, Knudsen force increases by approximately 104%, while rises by roughly 322% at Kn = 0.371. This means that the dependence of Knudsen force on $C_{0}$ is stronger in a high-pressure environment than in a low-pressure environment. Therefore, an appropriate enhancement of the working pressure can improve the sensitivity of the hydrogen sensors in practice. Evidently, based on the linear relation of Knudsen force to species concentrations, the calculation of approximate concentration of any species can be easy in binary mixtures, achieving the detection of species concentration in physical reality.

## 5. Concluding Remarks

In this study, the direct simulation Monte Carlo (DSMC) method is applied to investigate the effects of gas–surface interaction conditions (accommodation coefficients α, temperature, and carrier gas) on rarefied gas flows and hydrogen detection performance (Knudsen force) in MEMS sensors composed on triangle cold beams and rectangle hot beams at different pressures. The results demonstrate that gas flows and Knudsen force are strongly dependent on accommodation coefficients, temperature, and carrier gas. The Knudsen force nonlinearly increases as α decreases. Therefore, in order to achieve better detection performance, the application of special material to the device fabrication or the different treatments for the surfaces is extremely important. On the other hand, regarding the influence of working conditions, it is revealed that Knudsen force linearly depends on the temperature gradients ΔT, rising with the increase in ΔT. Specifically, when ΔT rises from 10 K to 300 K, the largest Knudsen force increases by approximately 28 times. In addition, a small Knudsen number Kn corresponds to a larger gas density; thus, the curve slopes for Knudsen force rise as Kn is reduced. This means that, when the gas flows tend to be in the transition regime, the dependence of Knudsen force on ΔT become more significant. This behavior can also be observed for the influence of carrier gas on Knudsen force. In addition, it is found that Knudsen force is almost a linear function of hydrogen concentration. Therefore, a correlation expression is proposed in this research for the application of the device to the detection of species concentration in practice. Based on the formula, the approximate species concentration in the H_2_-N_2_ mixtures can be easily calculated. In summary, the DSMC simulation results contribute to a better understanding of the dependence of hydrogen detection performance on gas–surface interaction conditions.

## Figures and Tables

**Table 1:** Pressure, temperature, and Knudsen number conditions used in the DSMC calculations.

$P_{0}\;(\mathrm{Pa})$	$T_{c}\;(K)$	$T_{h}\;(K)$	$Kn^{H_{2}}$	$Kn^{N_{2}}$	$Kn^{Mix}$
25	304.0	350.0	22.180	10.891	14.334
62	304.5	348.0	8.943	4.392	5.780
155	304.5	346.0	3.577	1.757	2.312
387	304.0	341.5	1.433	0.704	0.926
966	302.0	323.0	0.574	0.282	0.371

**Table 2:** Dependence of flow field and Knudsen force characteristics on the cell size, PPC, and time step in DSMC simulations at Kn = 0.371.

Test	Grid	Cells	PPC	Δt (ns)	$T_{avg}\;/\;T_{0}$	$\rho_{avg}\;/\;\rho_{0}$	$P_{avg}\;/\;P_{0}$	$U_{avg}\;(m/s)$	Knudsen Force (μN)
1	Coarse	10,942	20	1.0	1.218337	0.814183	1.010986	3.254461	25.37128
2	Standard	44,470	20	1.0	1.217954	0.813289	1.010958	3.219960	25.04571
3	Fine	177,880	20	0.1	1.217633	0.813224	1.010926	3.218653	25.02720
4	Standard	44,470	10	1.0	1.217935	0.813383	1.010891	3.226507	25.00109
5	Standard	44,470	50	1.0	1.218088	0.813211	1.010957	3.224241	25.03042
6	Standard	44,470	20	10	1.208874	0.824254	1.012472	3.387864	32.63538
7	Standard	44,470	20	0.1	1.218025	0.813298	1.010959	3.222712	25.06055

**Table 3:** Dependence of flow field and Knudsen force characteristics on the total simulation time in DSMC solutions at Kn = 0.371.

t (ms)	$T_{avg}\;/\;T_{0}$	$\rho_{avg}\;/\;\rho_{0}$	$P_{avg}\;/\;P_{0}$	$U_{avg}\;(m/s)$	Knudsen Force (μN)
7	1.217819	0.812434	1.010877	3.216330	25.04615
10	1.217954	0.813289	1.010958	3.219960	25.04571
15	1.218105	0.813435	1.010974	3.220644	25.04879
20	1.218023	0.813475	1.010941	3.221554	25.04794

**Table 4:** Effects of variations in surface accommodation coefficients of cold and hot beams on Knudsen force.

	Cold Beam				Hot Beam				Knudsen Force
	Bottom	Top	Left	Right	Bottom	Top	Left	Right	
Present	$\alpha_{n}=\sigma_{t}\;\mathrm{decrease}$		-	$\alpha_{n}=\sigma_{t}=1$	-	$\alpha_{n}=\sigma_{t}=1$			increase
Ref. [22]	$\sigma_{t}=1,\;\alpha_{n}\;\mathrm{decrease}$				$\alpha_{n}=\sigma_{t}=1$				increase
	$\alpha_{n}=1,\;\sigma_{t}\;\mathrm{decrease}$				$\alpha_{n}=\sigma_{t}=1$				decrease
Ref. [30]	$\sigma_{t}=1,\;\alpha_{n}\;\mathrm{decrease}$				$\sigma_{t}=1,\;\alpha_{n}\;\mathrm{decrease}$				decrease
	$\alpha_{n}=1,\;\sigma_{t}\;\mathrm{decrease}$				$\alpha_{n}=1,\;\sigma_{t}\;\mathrm{decrease}$				increase

## Data Availability

The data presented in this study are available on request from the corresponding author.
